# COVID-19: Mechanistic Model of the African Paradox Supports the Central Role of the NF-κB Pathway

**DOI:** 10.3390/v13091887

**Published:** 2021-09-21

**Authors:** Ralf Kircheis, Manfred Schuster, Oliver Planz

**Affiliations:** 1 Syntacoll GmbH, 93342 Saal an der Donau, Germany; 2 AGES GmbH, 1200 Vienna, Austria; schuster-manfred@a1.net; 3 Institute of Cell Biology and Immunology, Eberhard Karls University Tuebingen, 72076 Tuebingen, Germany

**Keywords:** SARS-CoV-2, COVID-19, cytokine storm, chemokine, NF-κB, African paradox, Duffy antigen receptor for chemokines (DARC), malaria incidence, helminth infection, macrophage polarization

## Abstract

The novel severe acute respiratory syndrome coronavirus 2 (SARS-CoV-2) has expanded into a global pandemic, with more than 220 million affected persons and almost 4.6 million deaths by 8 September 2021. In particular, Europe and the Americas have been heavily affected by high infection and death rates. In contrast, much lower infection rates and mortality have been reported generally in Africa, particularly in the sub-Saharan region (with the exception of the Southern Africa region). There are different hypotheses for this African paradox, including less testing, the young age of the population, genetic disposition, and behavioral and epidemiological factors. In the present review, we address different immunological factors and their correlation with genetic factors, pre-existing immune status, and differences in cytokine induction patterns. We also focus on epidemiological factors, such as specific medication coverage, helminth distribution, and malaria endemics in the sub-Saharan region. An analysis combining different factors is presented that highlights the central role of the NF-κB signaling pathway in the African paradox. Importantly, insights into the interplay of different factors with the underlying immune pathological mechanisms for COVID-19 can provide a better understanding of the disease and the development of new targets for more efficient treatment strategies.

## 1. Introduction

The novel coronavirus SARS-CoV-2 (severe acute respiratory syndrome coronavirus 2) was first reported in Wuhan, China, at the end of 2019 and has developed into the most serious global pandemic since the Spanish flu of 1918–1920, with more than 220 million affected persons and 4.6 million deaths worldwide by 8 September 2021 and 6-digit infection rates daily.

**Figure 1:**
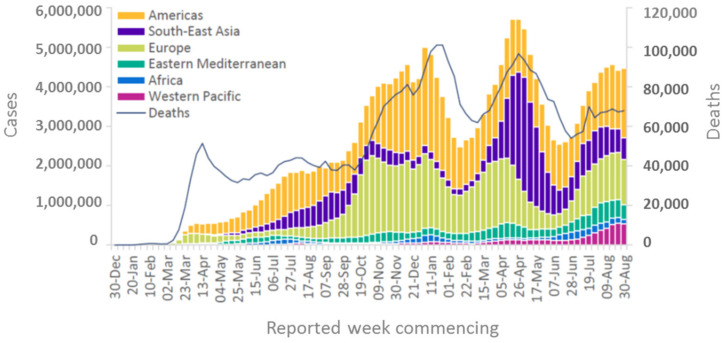
COVID-19 cases reported weekly by the WHO region and global deaths as of 30 August 2021. Source: WHO Coronavirus Disease (COVID-19) Weekly Epidemiological Update and Weekly Operational Update, Edition 56.

**Figure 2:**
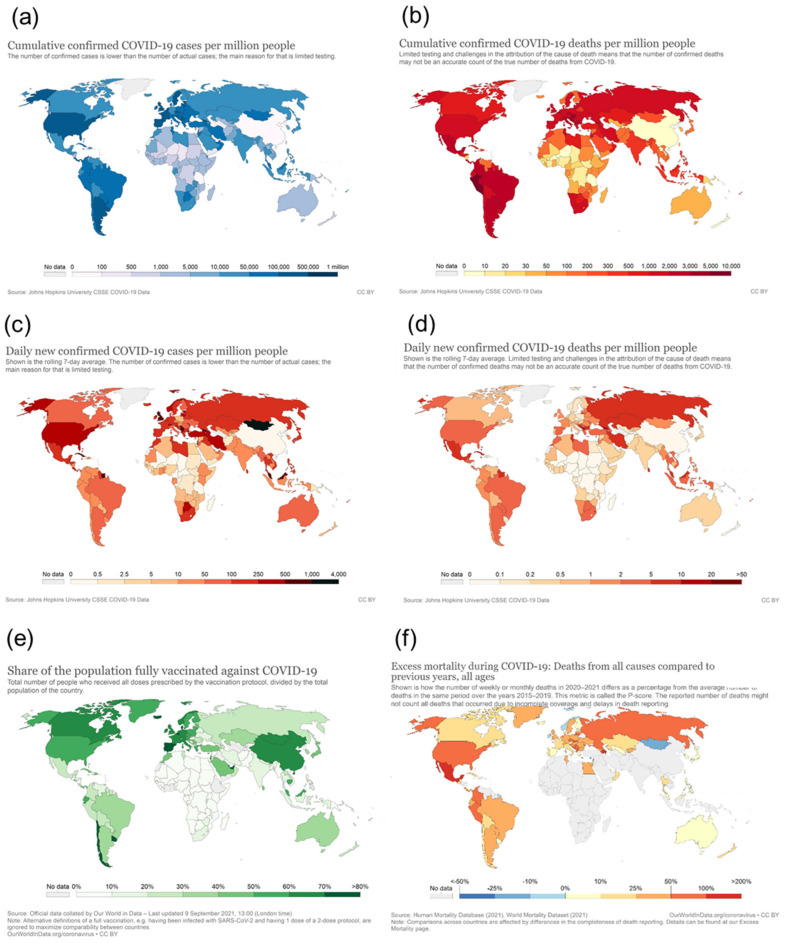
Cumulative confirmed COVID-19 cases per million people (**a**), cumulative confirmed COVID-19 deaths per million people (**b**), daily new confirmed COVID-19 cases per million people (**c**), daily new confirmed COVID-19 deaths per million people (**d**), share of the population fully vaccinated against COVID-19 (**e**), and excess mortality during COVID-19 (snapshot Jan 2021) (**f**). Source (**a**–**e**): https://ourworldindata.org/ (accessed on 8 September 2021).

When looking at the geographical pattern, North and South America and Europe have been affected most by COVID-19, with 85 mio infections (2.1 mio deaths) and 66 mio infections (1.3 mio deaths), respectively, as of 8th September 2021 (https://covid19.who.int/ accessed on 8 September 2021). In contrast, in Africa, a significantly lower infection rate and mortality have been reported (5.7 mio infections and 0.14 mio deaths) (Figure 1, compare area under each curve). Considering that Africa has a population of more than 1.3 billion (equivalent to 17% of the world population), which is more than that of the Americas (1.1 billion) and twice that of Europe (748 mio), Africa has very low numbers of SARS-CoV-2 infections and COVID-19-related deaths (2–3% of worldwide cases) (Figure 2a,b). Even though the number of new infections has been recently reported to increase, the total numbers are still low on the African continent compared to other parts of the world and are concentrated within a few countries located in Southern Africa, with this particular area accounting for more than 70% of new cases on the continent (Figure 2c,d, WHO weekly epidemiological update, Edition 50, 27 July 2021 [1]). Additional factors, such as the very low vaccination rate generally in Africa (approximately 1% of population fully vaccinated compared to more than 50% vaccination coverage in Western countries) (Figure 2e) and the high prevalence in Southern Africa of the SARS-CoV-2 delta and beta variants, with higher escape potential against some of the vaccine types, e.g., the ChAdOx1 nCoV-19 vaccine from AstraZeneca (which is one of the very few available vaccines for Africa), may drive the infection numbers up [2]. However, even including the current epidemiological factors, a significantly lower incidence and mortality rate for the African continent in general, and for the sub-Saharan area (except the Southern African area) in particular, are reported. While the incidence rate may be significantly under-represented by the low testing coverage on the continent, the mortality rate seems to be much lower in Africa compared to other parts of the world (see chapters below), and no excess mortality due to COVID-19 has been reported from African countries (Figure 2f). This is in sharp contrast to what had been expected considering Africa’s generally fragile health system, infrastructure, limited availability of trained medical personnel, and limited access to medical supplies and equipment [3,4]. Moreover, high population densities in African cities, with many people working in the informal business sector, make the implementation of strict look-down measures more difficult. Poverty and unhygienic conditions in rural areas are considered factors accelerating viral transmission [5]. This striking contrast to what had been expected, i.e., this “African COVID-19 paradox” [6], represents a very interesting piece in the puzzle of understanding COVID-19 disease and therapeutic approaches. There have been different hypotheses raised about these unexpectedly low infection and COVID-19 death rates in Africa, including a relative lack of testing and documentation, the younger age of the population, nutrition, more favorable climate, behavioral parameters, fewer comorbidities, and genetic disposition. From the present perspective, we address immunological mechanisms and their correlation with specifics of Africa’s population, genetic factors, pre-existing immune status, racial differences in cytokine induction patterns, high helminth infection rates in tropical regions, and specific factors related to malaria endemic to the sub-Saharan area, such as differences in Duffy antigen expression (DARC, Duffy antigen receptor of chemokines) and continent-specific coverage of various medications related to helminth infection and malaria.

## 2. Infectivity and Immunopathology Induced by SARS-CoV-2

SARS-CoV-2 belongs to enveloped positive-sense, single-stranded RNA viruses, similar to two other highly pathogenic coronaviruses, SARS-CoV and Middle East Respiratory Syndrome (MERS-CoV). SARS-CoV and MERS-CoV have been self-limiting or limited to sporadic endemic outbreaks, whereas the novel coronavirus SARS-CoV-2 has developed into a worldwide pandemic [7]. The reason for the highly efficient spread of SARS-CoV-2 compared to SARS-CoV and MERS-CoV may rely on its high binding affinity to the target receptor and the SARS-CoV-2 typical peculiarity of a long symptom-free but nevertheless highly infectious time period between infection and the appearance of first symptoms or asymptomatic transmission [8]. Both SARS-CoV and SARS-CoV-2 bind to the same receptor on the target cell, i.e., the angiotensin-converting enzyme-related carboxypeptidase-2 (ACE-2) receptor, by the highly conserved RBD of the spike (S) protein. The S protein is composed of the RBD containing S1 and the S2 subunit, which mediates fusion between the viral and host cell membranes after cleavage by the cellular serine protease TMPRSS2 [9]. This furin-like cleavage site is unique to the S protein of SARS-CoV-2 and may contribute to the higher infectivity of SARS-CoV-2. A more than 10-fold higher binding affinity to ACE2 of SARS-CoV-2 compared to SARS-CoV has been shown [10]. Various mutations have emerged, including the D614G mutation in the spike protein, which originally appeared in Europe and, until recently, has been the dominant circulating virus [11]. More recently, the SARS-CoV-2 variant B.1.1.7 (501Y. V1, alpha variant) with the N501Y spike protein mutation, observed initially in the United Kingdom [12,13], and further variants with multiple spike protein mutations, including the B.1.1.351 mutant (501Y. V2, Beta variant) (having N501Y—E484K—417N) first observed in South Africa [14], the P.1 (501Y. V3, gamma variant) (having N501Y—E484K –K417T), first reported from Brazil [15], and the B. 1. 617.2 (delta variant), first observed in India [16], appeared. They have an increased binding affinity and infectivity (e.g., due to the N501Y mutation) in common and partial escape from recognition from immune serum [2,12,16]. SARS-CoV-2 infection can affect pulmonary and multiple extra pulmonary tissues, including cardiac, kidney, and gastrointestinal organs, due to the broad expression of the ACE-2 receptor on pulmonary and cardiovascular tissues, monocytes, and tissue macrophages [9,17,18]. The interaction of SARS-CoV-2 with the ACE-2 receptor may also inhibit the lung-protective effect of the enzyme [19,20].

The majority of individuals infected with SARS-CoV-2 show mild-to-moderate symptoms, and up to 20% of infections may be asymptomatic. Symptomatic patients show a wide spectrum of clinical manifestations ranging from mild febrile illness and cough up to acute respiratory distress syndrome (ARDS), multiple organ failure, and death. Thus, the clinical picture of severe cases is very similar to that seen in SARS-CoV- and MERS-CoV-infected patients. While younger individuals show predominantly mild-to-moderate clinical symptoms, elderly individuals frequently exhibit severe clinical manifestations [21,22,23,24,25,26]. In addition to pre-existing comorbidities, including diabetes, respiratory and cardiovascular diseases, renal failure and sepsis, higher age and male sex seem to be associated with more severe disease and higher mortality [21,26,27,28,29]. Postmortem analysis showed diffuse alveolar disease with capillary congestion, cell necrosis, interstitial edema, platelet-fibrin thrombi, and infiltrates of macrophages and lymphocytes [30]. Furthermore, the induction of endotheliitis in various organs (including lungs, heart, kidney, and intestine) by SARS-CoV-2 infection as a direct consequence of viral involvement and of the host inflammatory response was shown [9,17,18].

The molecular mechanisms for the morbidity and mortality of SARS-CoV-2 are still incompletely understood. Virus-induced cytopathic effects and viral evasion of the host immune response are assumed to play a role in disease severity. However, clinical data from patients, particularly those with severe clinical manifestations, indicate that highly dysregulated exuberant inflammatory and immune responses correlate with the severity of disease and lethality [17,18,22,30,31,32,33]. Significantly elevated cytokine and chemokine levels, also termed “cytokine storm”, play a central role in severity and lethality in SARS-CoV-2 infections. Elevated plasma levels of IL-1β, IL-7, IL-8, IL-9, IL-10, G-CSF, GM-CSF, IFNγ, IP-10, MCP-1, MIP-1α, MIP-1β, PDGF, TNFα, and VEGF have been reported. Significantly higher plasma levels of IL-2, IL-7, IL-10, G-CSF, IP-10, MCP-1, MIP-1α, and TNFα were found in patients with severe pneumonia developing ARDS compared to non-ICU patients showing pneumonia without ADRS [22]. Various studies have shown that highly stimulated epithelial–immune cell interactions lead to exuberant dysregulated inflammatory responses with significantly (topically and systemically) elevated cytokine and chemokine release [34,35]. Furthermore, recent data have indicated that the NF-κB pathway is a central signaling pathway for the SARS-CoV-2 infection-induced pro-inflammatory cytokine/chemokine response, playing a central role in the severity and lethality of COVID-19 [36,37,38,39,40,41,42]. Within endosomes, SARS-CoV-2, as a single-stranded RNA virus, is assumed to activate the Toll-like receptors TLR7 and TLR8. Furthermore, double-stranded RNA intermediates generated during viral replication can be recognized by TLR3. TLR7/8 or TLR3 activation leads to activation of the transcription of the interferon-regulator factor (IRF) family and antiviral responses. However, the activation of the TLR also triggers—via various intermediates—the activation of IKK (IκB kinases), leading to phosphorylation of the cytoplasmic inhibitor factor IκBα followed by its ubiquitination and degradation by the 26S proteasome. Thereby, NF-κB (a heterodimer complex of subunits p50 and p65) is released from IκBα and can enter the nucleus and initiate the transcription of various genes coding for cytokines, chemokines, adhesion molecules, and growth factors. This NF-κB-triggered pro-inflammatory response in acute COVID-19 is shared with other acute respiratory viral infections caused by highly pathogenic influenza A virus of H1N1 (e.g., Spanish flu) and H5N1 origin, SARS-CoV and MERS-CoV [42]. Excessive NF-κB activation and exuberant inflammatory responses with the involvement of endothelial cells, epithelial cells, and immune cells lead to further disturbances in other integrated systems, including the complement system, coagulation, and bradykinin systems, and feeding back into positive signaling feedback loops, accelerating COVID-19-associated inflammatory processes [43,44,45,46,47,48].

## 3. Reasons for Lower Mortality and Morbidity of COVID-19 in Africa Compared to Other Regions of the World

In the following sections, the various socioeconomic, environmental, genetic, epidemiological, and immunological factors and their interplay are addressed.

### 3.1. Situation of Testing and Reporting of SARS-CoV-2 Infections and COVID-19 Cases

**Figure 3:**
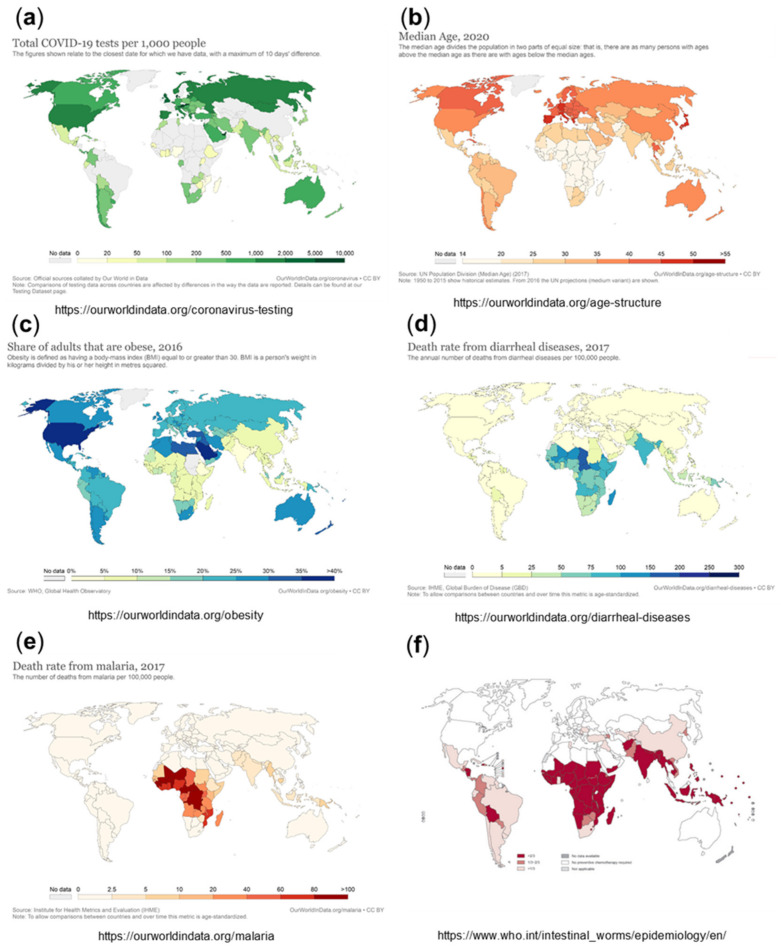
Total COVID-19 tests per 1000 people, from July 2021 (**a**), medium age, 2020 (**b**), share of adults who are obese, 2016 (**c**), death rate from diarrheal diseases (**d**), death rate from malaria, 2017 (**e**), and intestinal worms/epidemiology/soil transmitted helminthiases, 2011 (**f**). Sources (**a**–**e**): https://ourworldindata.org/ accessed on 5 July 2021; (**f**) https://www.who.int/intestinal_worms/epidemiology/en/ accessed on 5 July 2021.

Overall testing capacities for SARS-CoV-2 have been limited in Africa compared to other parts of the world. With the first case of COVID-19 recorded in Africa in February 2020, on the whole continent, there were only a few institutes in place for COVID-19 testing; meanwhile, all African countries are in the position to diagnose COVID-19 [4,5]. The testing rates in Africa are still generally lower than those in leading industrial or wealthy countries, such as the USA, Europe, Russia, the United Arab Emirates, Australia, and Canada, although not so different from those in other parts of the world, including Iran, India, Pakistan, Indonesia, and South and Middle America [49]. Setting the cumulative number of tests in relation to the confirmed cases shows even fewer differences [5]. Nevertheless, the absolute numbers of tests per 100,000 population are lower than those outside Africa (Figure 3a), which is expected to lead to significantly under-estimated numbers for the infection rates, with a high number of undetected (asymptomatic or with mild symptoms) infections. Studies suggest that the asymptomatic spread of the disease has been significantly more widespread than the numbers may indicate [50]. This is illustrated by a recently published study, which determined the seroprevalence of anti-SARS-CoV-2 IgG to be 5.6% among blood donors in Kenya in April–June 2020. The population-weighted national seroprevalence was 4.3% and was particularly high in the three urban counties, Mombasa (8.0%), Nairobi (7.3%), and Kisumu (5.5%), indicating a much higher than expected population exposure across Kenya. The SARS-CoV-2 seroprevalence in this study was comparable with those in Europe, Brazil, and the United States. Based on an estimated population of 53 million in Kenya, with 57% of the population aged 15 to 64 years, the authors calculated that if applying the transfusion donor seroprevalence of 4.3% to all 15- to 64-year-olds, it would suggest 1.3 million infections. However, in the same period, only 2093 cases were detected (with 90% asymptomatic), and there were only 71 deaths among all ages, implicating an apparently much lower mortality and morbidity of COVID-19 in this African country compared to other parts of the world [51]. 

This was even more dramatically illustrated by a recent study published as a Lancet Preprint. Of 1164 individuals from 527 households tested, the seroprevalence was 34.7%, indicating that roughly 1.5 million Nairobi residents had been infected. Half of the households had at least one positive participant. The Infection Fatality Ratio (IFR) was 40 per 100,000 infections. Over one third of Nairobi residents in half of the households were infected by November 2020, indicating extensive transmission in the city, comparable to countries reporting more severe forms of the pandemic. However, the IFR was more than 10-fold lower than that reported in Europe and the United States, supporting the perceived low morbidity and mortality in sub-Saharan Africa [52]. What could be the reasons for this lower mortality and morbidity of COVID-19 in Africa?

### 3.2. Previous Experience in Epidemic Control from Tackling Other Infectious Diseases

In contrast to the initially very limited test capacities, epidemic control measures were very swiftly implemented by most African countries. From their experience with endemics of highly infectious deadly viruses such as Ebola virus, many African countries focused very early on preventing COVID-19 importation, implementing enhanced surveillance at airports and suspending direct flights to and from China [3]. International and public travel closures had already been implemented by the end of March 2020, i.e., earlier than many Western countries. Furthermore, immediately after confirmation of the first cases on the continent, most African governments took drastic public health measures to slow down the spread of the virus, including isolation of infected persons and quarantining their contacts. Furthermore, due to experience with Ebola outbreaks, many African citizens are accustomed to behavioral adaptations, such as the elbow bump, frequent hand washing, and the need for masking. This could have resulted in lower viral transmission rates [3].

Additional factors for lower viral transmission rates are lower travel rates due to less developed transport systems, lack of sightseeing habit history, and more outdoor living, where infectious respiratory droplets and aerosols are more easily dispersed.

### 3.3. Impact of the Climate on the Spread and Progression of SARS-CoV-2

Various infectious diseases, including human CoVs, show seasonal patterns in their incidence. Environmental factors such as temperature and humidity were shown to impact the spread of infections for SARS-CoV, with significantly reduced virus viability at higher temperatures and relative humidity (38 °C and >95% RH). Moreover, SARS-CoV-2 more easily spreads at lower temperatures and humidity, with an increase of 1 °C in temperature correlating with a decrease in the prevalence of COVID-19 by 5.4% [53]. A meta-analysis showed that cold and dry conditions potentiate viral spread, while warm and wet climates seem to reduce the spread of SARS-CoV-2 [54,55]. The warm and tropical climate could therefore have some contribution to the lower morbidity and mortality found in sub-Saharan Africa, although this effect should be limited, as indicated by the high morbidity and mortality seen in the subtropical regions of South America.

### 3.4. Specifics of Africa’s Population

#### 3.4.1. Correlation with Age and “Inflamm-Aging”

One important factor contributing to the lower mortality and morbidity of COVID-19 in Africa is probably its relatively young population, with a median age of 19.7 years (see also Figure 3b), with more than 60% being under the age of 25 and only approximately 3% of the population aged over 65 years [5,56]. In comparison, Europe, North America, and wealthier Asian countries have the oldest populations (see Figure 3b).

There is a well-recognized correlation between age and severity of COVID-19, with most of those who have died being aged over 80 [26,27,28]. The higher vulnerability of aged persons to COVID-19 is probably related to accumulated comorbidities and a generally higher level of immune activation and inflammation in elderly people. Multiple reports have shown that the severity of COVID-19 infections varies widely from children (often asymptomatic), adults (mostly mild infection), and elderly adults (frequently deadly critical). The COVID-19-induced cytokine storm, i.e., severe systemic elevation of pro-inflammatory cytokines, has been more frequently found in elderly persons than in young adults and children [21].

In this context, aging is generally associated with elevated levels of local and systemic pro-inflammatory cytokines, creating a chronic condition of inflammation in aged persons, also termed “inflamm-aging”. Multiple studies have shown elevated levels of IL-6, IL-1, TNFα, and C-reactive protein (CRP) in aged persons. Therefore, it is likely that dysregulation of cytokine homeostasis in “inflamm-aging” persons may play a critical role in the risk of a cytokine storm and subsequently acute respiratory distress syndrome (ARDS) or multiorgan failure found in elderly persons with acute COVID-19. Furthermore, it seems that the “cytokine storm” phenomenon in elderly patients with severe COVID-19 is also associated with many age-related pathophysiologic processes, such as alteration of angiotensin-converting enzyme 2 (ACE2) receptor expression, excess ROS production, alteration of autophagy, inflammatory cell activity—particularly in adipose tissue—immune senescence, and lack of vitamin D [57].

Regarding the underlying molecular mechanism, one central signal transduction pathway, NF-κB, has widely been accepted as one of the key pathways involved in aging. Among the various types of cellular damage contributing to aging, genotoxic, inflammatory, ER stress, and oxidative stress have been shown to stimulate the NF-κB pathway. Various biological mechanisms implicated in aging, such as immune responses, cell senescence, apoptosis, and metabolism, are regulated at least in part by NF-κB. Additionally, other cellular processes implicated in regulating life span, including insulin/IGF-1, growth hormone pathways, SIRT, FoxO, and mTOR, are all interconnected with the NF-κB pathway [58]. Notably, using motif mapping, NF-κB was determined to be the transcription factor most associated with aging [59]. Increased basic levels of inflammation, with upregulated NF-κB signaling and cytokine secretion, have been found in age-associated diseases such as atherosclerosis, osteoporosis, neurodegeneration, diabetes mellitus type 2 (referred to as ‘diabetes’ in the forthcoming sections), and cardiovascular disease [58,60,61,62,63]. Chronically elevated activity levels of NF-κB during aging and age-associated diseases [57,58,60,61,62,63] can be expected to act additively or synergistically (via positive feedback loops), increasing SARS-CoV-2-triggered NF-κB activation [41,42], leading to massive cytokine and chemokine release and increased expression of activation markers and adhesion molecules. Furthermore, downregulation of ACE2 following binding of SARS-CoV-2 might lead to an accumulation of its substrate Ang II, which is known to activate the NF-κB signaling pathway [64].

Furthermore, the low percentage of elderly people living in nursing homes may contribute to the low mortality in Africa. In contrast to Western countries, elderly homes are relatively rare in most African countries, where older people are more likely to live in rural areas or to return to their rural homes when they retire [65]. Importantly, a large proportion of reported COVID-19-associated deaths in Western countries are attributable to outbreaks in nursing homes for elderly individuals [12].

#### 3.4.2. Low Prevalence of Comorbidities in the African Population

Another specific of Africa’s population is that some typical diseases of the Western population, such as obesity and diabetes, have an apparently lower incidence in Africa than in Western countries (see Figure 3c) and are particularly rare in Africa’s young population. Various comorbidities, such as hypertension, diabetes, respiratory diseases, cardiovascular diseases, cerebrovascular diseases, kidney diseases, and cancer, have been demonstrated to correlate with significantly higher mortality in COVID-19 patients [66]. In particular, regarding obesity and diabetes, there is growing evidence suggesting that obese patients face severe COVID-19 symptoms more frequently [28,29,66,67]. In this context, the significantly lower rate of obesity and related diabetes certainly contributes to the low mortality rate of COVID-19 in Africa.

Regarding the underlying molecular mechanisms, recent studies have revealed that a fructose-rich diet triggers inflammation and lipid synthesis via sterol regulatory element binding protein-2 (SREBP-2) activation. Altered cholesterol and lipid synthesis were associated with NLRP and NF-κB activation and increased levels of the proinflammatory cytokines IL-1β and TNFα. Furthermore, obese individuals had increased levels of endotoxin in the plasma (due to disturbances in intestinal barrier integrity) [68], leading to TLR4- and NF-κB-mediated inflammatory cytokine production, including IL-1β, TNFα, and IL-6 [29,69]. Multiple in vitro and animal studies indicate that NF-κB activation is a key event early in the pathobiology of diabetes. Prolonged hyperglycemia was shown to induce NF-κB activation in type I and II diabetes. Several mechanisms are involved in NF-κB pathway activation, such as hyperglycemia-induced IKKβ overexpression, increased Protein kinase C (PKC) activity, and the formation of advanced glycosylation end products (AGEs), which bind to specific receptors, i.e., receptors for advanced glycosylation end products (RAGE) on vascular smooth muscle cells, leading to oxidant stress, i.e., reactive oxygen species (ROS), and increased adhesion molecules, such as ICAM-1 and vascular cell adhesion molecule-1 (VCAM-1). NF-κB activation was accompanied by increased cytokine expression, inducing positive feedback loops via TNFα/IL-1β receptor-triggered NF-κB activation and increased expression of RAGE. In addition to TNFα and IL-1β, IL-6, IL-8, and MCP-1 are associated with the pathogenesis of diabetic microvascular and macrovascular complications such as cardiomyopathy, retinopathy, nephropathy, and neuropathy [70,71,72,73]. Recent reports have indicated that the NF-κB pathway acts as a central signaling pathway for the SARS-CoV-2 infection-induced pro-inflammatory cytokine/chemokine response and plays a central role in the severity and lethality of COVID-19 [41,42]. Accordingly, chronically pre-elevated NF-κB activity levels in diabetic people may act additively or synergistically, increasing SARS-CoV-2-triggered NF-κB activation [42] and resulting in massive cytokine and chemokine release and activation marker and adhesion molecule expression [74].

#### 3.4.3. Genetics and Specifics of the Immune System

A genome-wide association study of severe COVID-19 pathological manifestations with respiratory failure identified two genomic regions associated with severe courses of COVID-19: one region on chromosome 3 (locus 3p21.31) containing six genes and one region on chromosome 9 (9q34.2) that determines ABO blood groups. The region on chromosome 3 includes a cluster containing genes encoding chemokine receptors, such as CC motif chemokine receptor 9 (CCR9) and C-X-C motif chemokine receptor 6 (CXCR6). Moreover, the flanking genes (CCR1 and CCR2) code for chemokine receptors involved in inflammatory responses [75,76].

Zeberg and Pääbo have shown that the genetic variants most associated with severe courses of COVID-19 on chromosome 3 span a region of almost 50 kilo bases and are all strongly associated with each other within the population (r2 > 0.98). Interestingly, this region is carried by ~50% of people in South Asia and ~16% of people in Europe today but is practically absent in African populations. The study demonstrated that the risk haplotype has entered the modern human population by gene flow from Neanderthals. Notably, Neanderthal-derived haplotypes are almost completely absent in the African population, consistent with the very limited gene flow from Neanderthals into African populations. In addition to other risk factors, the Neanderthal haplotype has been suspected to be a contributor to COVID-19 risk in certain populations [77]. Furthermore, 2–8% of people in Eurasia carry a variant promoter region of the DPP4 gene inherited from Neanderthals. This gene encodes the enzyme dipeptidyl peptidase IV, which serves as a receptor for MERS-CoV but not for SARS-CoV-2. Nevertheless, the Neanderthal DPP4 variant was calculated to double the risk of becoming critically ill in COVID-19. The Neanderthal DPP4 haplotype is present in ~1% of Europeans, ~2.5% of South Asians, ~4% of East Asians, and ~0.7% of admixed Americans. It is absent among Africans south of the Sahara [78].

#### 3.4.4. HLA Variability

Another potential genetic contributor to the lower incidence of COVID-19 in Africa may be the prevalence of different human leucocyte antigen (HLA) alleles in Africa compared to other regions. HLA alleles, particularly major histocompatibility complex (MHC) class I alleles, are essential for the presentation of viral antigens and thus define viral resistance and disease intensity. Specific HLA genotypes can stimulate the T-cell-mediated antiviral response differently and can thereby alter the symptoms and transmission of the disease. The HLA system was shown to affect clinical outcomes in multiple infectious diseases, including SARS, with demonstrated correlations between certain HLA alleles and the incidence and severity of SARS [79].

In silico analysis of viral peptide–MHC class I binding affinity revealed that certain specific HLA haplotypes effectively presented a larger number of SARS-CoV-2 peptides than other haplotypes, e.g., HLA-B*15:03 demonstrated the greatest ability to present highly conserved SARS-CoV-2 peptides shared among common human coronaviruses, indicating that this allele may allow cross-protective T-cell-dependent immunity. This is intriguing, as HLA-B*15:03 is prevalent in West Africa and most countries with high endemic malaria in the African region [79,80].

In this context, the correlation of HLA haplotype distribution and the incidence of malaria presents another potentially interesting mechanism. HLA-B27 seems to confer susceptibility to malaria since HLA-B27 prevalence is lower in malaria-endemic populations [81]. A correlation of HLA-mediated host peptide presentation with COVID-19 disease was hypothesized with respect to the lower COVID-19 incidence numbers in malaria-endemic populations. In this context, a correlation with HLA-B*27:07 was found in patients with a severe course of COVID-19 [82]. Further research will be necessary for a more comprehensive picture of the potential associations of HLA haplotypes and susceptibility to severe courses of COVID-19.

#### 3.4.5. Differences in Cytokine Responses for Africa-Derived Persons Compared to Persons from Other Continents

A further aspect relates to COVID-19 immunopathology and different cytokine responses for Africa-derived people compared to people from other continents. The association of elevated plasma IL-18 with the incidence of diabetes was studied in a 9-year follow-up of 9,740 middle-aged, initially healthy, nondiabetic white and African American individuals (50% white, 50% African American), random samples of both cases of incident diabetes (*n* = 548), and eligible members of the full cohort (*n* = 536). Notably, baseline IL-18 levels were significantly higher in white participants than in African American participants and correlated with age, anthropometric characteristics, and adiponectin. The study suggested that there are racial differences in the levels of IL-18 and the association of IL-18 with risk factors for diabetes [83]. Notably, IL-18 expression is dependent on NF-κB signaling, and IL-18 has been shown to correlate with the severity and mortality of COVID-19 [84].

The innate cytokine response following pattern recognition receptor (PRR) stimulation of whole blood from 2-year-old infants across four continents, i.e., from Africa, North America, South America, and Europe, was studied. The innate cytokine responses were rather similar for infants from North America, South America, and Europe. In contrast, PBMCs from African infants secreted significantly lower levels of cytokines following stimulation of extracellular and endosomal (but not after stimulation of cytosolic) pattern recognition receptors (PRRs). In particular, for endosomal responses, such as following the stimulation of TLR7/8 or TLR3 or after stimulation of the cell-surface-expressed TLR4 by LPS, significant differences in the cytokine release patterns were found, including the pro-inflammatory cytokine pattern (TNFα, IL-1β, CXCL8, CCL3, CCL4), the Th1 cytokine pattern (IFNα2, IFNγ, CXCL10, IL-12p70), and the Th17 cytokine pattern (IL-12p40, IL-6), with significantly lower expression from cells from infants from Africa compared to the other three sites. In contrast, the cytokine pattern released in response to cytosolic PRR (TLR2/1 and NOD2) was similar for all four groups, indicating that there is not an overall inability of African infants for cytokine production to PRR stimulation but that there are variations in the response to particular types of PRR stimulation [85].

Furthermore, significantly lower intracellular cytokine production in monocytes and dendritic cells together with a markedly lower polyfunctionality in African children compared to children from other continents was shown. The number of cells expressing two or three different cytokines (e.g., TNFα + IL-6) compared to single-cytokine-expressing cells was significantly lower in African children [86]. Single-nucleotide polymorphisms within the PRR pathways and differences in the prevalence of these single-nucleotide polymorphisms may contribute to the differences in functional responses [87]. Furthermore, the authors hypothesized that the particular constellation of microbiota in African infants may contribute to the observed difference in innate immune phenotypes [85,86,87]. Together, the lower IL-18 expression in African Americans [83] and the lower cytokine release upon PRR stimulation in African children may be indicative of lower stress-induced cytokine expression in African people, which in turn may promote a more controlled inflammatory response, contributing to lower morbidity and mortality for COVID-19 in Africa.

### 3.5. Epidemiology and Exposure to Different Pathogens

While non-communicable diseases, such as obesity and diabetes, have a lower incidence, infectious diseases such as HIV, tuberculosis, malaria, diarrheal diseases, and infections by helminths (parasitic worms) are highly prevalent in Africa (Figure 3d–f). It is suspected that regular exposure to different pathogens, including various other coronaviruses, may confer some level of resistance to COVID-19. Antibodies or T cells stimulated by previous encounters with human coronaviruses that cause “common cold” might cross-react and neutralize SARS-CoV-2 in humans. Pre-existing cross-reactive T cells can be found in individuals who have not been exposed to SARS-CoV-2, suggesting that previous exposure to related coronaviruses could have generated a certain level of cross-reactivity, most likely due to peptides derived from viral proteins that are conserved between different coronavirus strains [88,89].

Generally, it is increasingly recognized that the immune system is shaped not only by genetic disposition but also by environmental factors, such as exposure to microorganisms and parasites. This educates the immune system to protect against invading pathogens not only in a specific manner but also nonspecifically through, e.g., “trained immunity”, which may involve reprogramming of innate cells, which, upon secondary encounter with a pathogen, can deliver a stronger and faster response [3]. In this context, early chronic exposure to pathogens may induce strong regulatory immune responses that counteract excessive inflammation [90]. A study has shown that monocytes of African individuals with high exposure to pathogens are less pro-inflammatory, as measured in the context of the yellow fever vaccine YF-17D, in a volunteer cohort from Uganda compared to a cohort from Switzerland [91].

#### 3.5.1. High Incidence of Helminth Infection in Sub-Saharan Africa and Their Potential Effect on COVID-19

There is a potential correlation of lower COVID-19 incidence with the high prevalence of infections with helminth parasites in Africa [92]. Helminths are known to skew the host immunity towards type 2 responses characterized by cytokines such as IL-4, IL-5, IL-9, and IL-13, produced by Th2 cells and alternatively activated macrophages (M2). Moreover, helminths have evolved efficient approaches for immune suppression, such as the generation of regulatory T cells (Treg) and stimulation of the release of anti-inflammatory cytokines IL-10 and transforming growth factor-β (TGF-β). This represents an effective strategy for subverting protective immune responses to prolong the survival of the helminths in the host, which, however, also has bystander effects of modulating immune responses to unrelated antigens. Epidemiological studies have shown that infection with helminth parasites is associated with a lower incidence of allergies, asthma, and autoimmunity in developing countries. Studies in mice have demonstrated that regulatory immune responses induced by helminth can suppress both Th2 and Th1/Th17 responses that mediate allergy and autoimmunity, respectively [92,93].

Moreover, helminth-induced alterations of the gut microbiome have been shown to mediate systemic immunomodulatory effects [94,95]. Helminth coinfection was shown to influence the severity of viral infection in mice. Regarding murid herpesvirus (MuHV-4) respiratory infection, prior infection with *Schistosoma mansoni* reduced disease severity [96]. Infection of mice with *Fasciola hepatica* induced IL-10 release, which subverted parasite-specific Th1 and Th2 responses and induced TGF-β to suppress autoantigen-specific Th1 and Th17 responses involved in autoimmune diseases [97]. 

Another line of trained immunity is the so-called “virtual memory” mediated by bystander or virtual memory CD8+ T cells (TVMs), which represent a subset of nonconventional T cells displaying memory properties that can be generated through responsiveness to IL-4 rather than through pathogen-specific antigens. Helminth-induced type 2 immunity was shown to expand TVM cells through direct IL-4 signaling to CD8+ T cells. Conditioning of TVM cells provided enhanced control of acute respiratory infection with MuHV-4, which correlated with an increase in antigen-specific CD8+ T cell effector responses in the lung [98]. Helminth-induced IL-5 and IL-33 were shown to provide protection against autoimmunity [99]. These studies demonstrate that helminths can modulate the host immune response to reduce protective immunity and repulsion of helminths, but this is accompanied by significant bystander effects on specific and nonspecific responses to other targets, amelioration of Th1 and Th17 immunopathology, and can even induce bystander memory effects to unrelated antigen-specific CD8+ T cells.

#### 3.5.2. Role of M1 Th1 vs. M2 Th2 Polarization for COVID-19

In this context, helminth-induced cytokines, e.g., IL-4, and their impact on M1–M2 macrophage and Th1–Th2 T helper cell polarization may be of particular interest. While M1-like macrophages are essential for the initial inflammatory responses, M2-like macrophages are critical for tissue repair after pathogen clearance. M1-like macrophages are associated with microbicide activity, pro-inflammatory cytokine production, and the immune response. They are characterized by the production of inflammatory mediators, reactive oxygen species, antigen presentation, phagocytic activity, and the expression of MHC II molecules [100]. Usually, the recognition of pathogen-associated molecular patterns confers an M1-like phenotype associated with the release of inflammatory cytokines (IL-6, TNF-α, and IL-12) and interferons, which stimulate Th1 cell activity. Th1 cells release IFN-γ, TNF-β, and IL-2, activating macrophages and phagocyte-dependent responses [101].

After the inflammatory response is unfolded, there is normally a switch to the M2-like phenotype to initiate the resolution of inflammation, maintain tissue integrity, and return to homeostasis, characterized by decreased production of inflammatory mediators and the release of anti-inflammatory cytokines such as IL-10 or TGF-β [102]. M2-like macrophages secrete anti-inflammatory cytokines and play essential roles in angiogenesis, vascularization, immunosuppression, and immune tolerance [103,104]. Release of TGF-β promotes ECM deposition, wound healing, and scar formation, which can have negative long-term effects, e.g., resulting in fibrosis and cirrhosis [105,106]. A powerful imbalanced M1-like polarization can, at later time points, also stimulate the activity of M2-like macrophages. M2-induced proliferation and activation of fibroblasts and secretion of collagen and other ECM components will exacerbate the formation of fibrotic tissue, potentially leading to long-lasting, harmful effects [107]. With regard to COVID-19, some studies have indicated a mixed M1/M2 phenotype in circulating monocytes following SARS-CoV-2 infection [108,109]. However, the cytokine/chemokine pattern most characteristic of COVID-19, i.e., TNFα, IL-1, IL-6, IL-12, MCP-1, CXCL10, represents—at least in the acute stages—a typical M1–Th1 pattern [110], which may be accompanied by partial M2–Th2 responses (i.e., IL-10) during later phases [111] as a reactive repolarization of macrophages over the time course of inflammatory reactions [100]. In the context of COVID-19, frequently, increased levels of IL-10 are reported, usually at later stages. Notably, the IL-10-induces the M2c phenotype, inhibiting both Th1 and Th2 responses [112]. Prominent M1 polarization in BALF with significant CXCL9, CXCL10, and CXCL11 concentrations has been demonstrated in severe and moderate COVID-19 patients [113].

M1 macrophage polarization associated with pro-inflammatory cytokines TNFα, IL-1, IL-6, and IFNγ is related to signal transduction pathways, such as the AP1, NF-κB, and STAT1 pathways. While COVID-19 is characterized by an IFNγ deficiency or at least delayed responsiveness [114,115], NF-κB pathway activation seems to be central to the severe disease stage of COVID-19 [41,42]. Interestingly, a suppressing effect of IL-4Ra receptor-mediated M2 polarization on NF-κB-induced M1 polarization has been shown [112,116]. Moreover, p50—a major negative regulator of the NF-κB canonical pathway—is involved in M2 macrophage polarization processes [117]. Taken together, these data indicate that NF-κB triggers M1 polarization in acute-stage COVID-19, with prominent M1/Th1 cytokine and chemokine release, which may partially be counterbalanced in the case of helminth-induced M2/Th2 polarization.

#### 3.5.3. Treatment of Helminth Infections with Ivermectin and Its Effect on COVID-19-Triggered Cytokine Storm

In addition to the immunomodulatory effect of helminth infection on COVID-19 susceptibility, there is another observation linked to a compound used for the treatment of helminth infections, i.e., ivermectin [118]. There was one early study that indicated that countries with routine drug administration of prophylactic chemotherapy (PCT), including ivermectin, in various African countries correlated with a significantly lower COVID-19 incidence than countries where no prophylactic chemotherapy or PCT without ivermectin was used [119]. In vitro studies showed inhibition of the replication of SARS-CoV-2 by ivermectin, although only at therapeutically non-relevant high doses [120]. 

Interestingly, ivermectin (or avermectin) showed anti-inflammatory effects by downregulating the NF-κB and MAP kinase pathways. Avermectin inhibited the LPS-induced release of TNFα, IL-1β, and IL-10 by inhibiting NF-κB p65 translocation into the nucleus and JNK and p38 phosphorylation in vitro, improving the survival of mice [121,122]. 

Anecdotally, the use of the standard clinical dose of ivermectin has been associated with some cases of rapid clinical resolution in severely hospitalized COVID-19 patients. [123]. A meta-analysis assessing a total of 629 SARS-CoV-2-positive patients from four studies suggested that the primary outcome, i.e., all-cause mortality, was decreased almost by half (odds ratio 0.53) [124]. However, more recent data from a double-blind randomized trial showed no significant effects and concluded that, for adults with mild COVID-19, a 5-day course of ivermectin did not significantly improve the time to resolution of symptoms [125]. 

#### 3.5.4. High Incidence of Malaria Infection in Sub-Saharan Africa and Their Potential Effect on COVID-19

**Figure 4:**
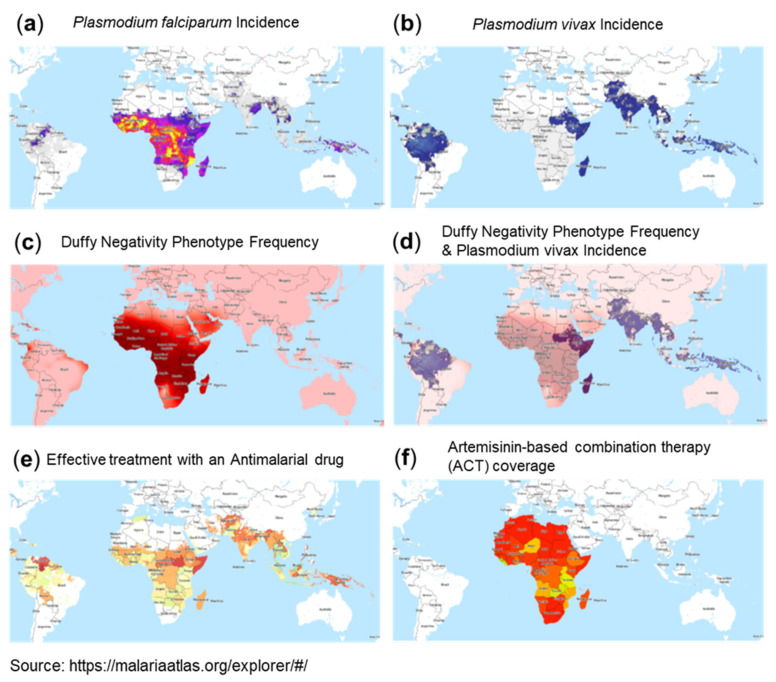
*Plasmodium falciparum* incidence (**a**), *Plasmodium vivax* incidence (**b**), Duffy negativity phenotype frequency (**c**), Duffy negativity phenotype frequency and *Plasmodium vivax* incidence overlay (**d**), Effective treatment with an antimalarial drug (**e**), and Artemisinin-based combination therapy (ACT) coverage (**f**). Source: Malaria Atlas Project, https://malariaatlas.org/explorer/#/ accessed on 5 July 2021. Note: high incidence regions of the two major plasmodium species, *Plasmodium falciparum* and *Plasmodium vivax*, partially overlap; however, they show large discrepancies in sub-Saharan Africa despite similar climatic needs. This may be considered a consequence of the high level of Duffy negativity in the sub-Saharan African population, which serves as the entry receptor for *Plasmodium vivax* into erythrocytes, whereas *Plasmodium falciparum* uses multiple entry receptors. Among the countries with effective treatment with anti-malaria drugs, only Africa has high ACT coverage.

A most interesting observation is the correlation between malaria incidence and reported low numbers for COVID-19. Malaria affects hundreds of millions of people and causes approximately 440,000 deaths each year. Malaria is distributed as a worldwide malaria-endemic belt in the equatorial and tropical zones (Figure 3e and Figure 4a,b). Interestingly, epidemiological studies have indicated that the endemic presence of malaria seems to protect populations from the COVID-19 outbreak, particularly in less developed countries. Moreover, similar to SARS-CoV-2, historical data of SARS-CoV and MERS-CoV epidemics showed significantly lower incidences in countries of the worldwide malaria-endemic belt [126,127]. This inverse correlation between incidences of malaria infections and COVID-19 could be due to: (1) the presence of an evolutionary adaptation related to malaria in endemic areas that provides an advantage against COVID-19 or (2) the effect of therapies used for malaria treatment or prophylaxis.

One aspect that could be related to susceptibility to COVID-19 is genetic polymorphisms linked to susceptibility to malaria. *Plasmodium falciparum* malaria is a major cause of mortality and morbidity and has imposed strong selective forces on the human genome in endemic regions of sub-Saharan Africa (Figure 3e and Figure 4a). Host genetic factors are assumed to contribute to the susceptibility to *Plasmodium falciparum* infection, which may potentially also have an impact on COVID-19 severity. Several gene mutations and polymorphisms confer survival advantages and have increased in frequency through natural selection in endemic areas. These include sickle cell traits (HbAS) and hemoglobinopathies such as thalassemias and glucose-6-phosphate dehydrogenase (G6PD) deficiency. A panel of gene polymorphisms has been identified that appear to affect malaria susceptibility by modulation of the immune response or by interfering with host–parasite interactions, including HLA-B, HLA-DRB1, TNFα, IFNγ, IL-1α,β, IL-4, IL-10, IL-22, TLR4,1,6,9, ICAM-1, and complement receptor CR1. A neutrophil-related gene region contained genes encoding mediators of innate and adaptive immunity, including those for cytokine receptors, Toll-like receptors, heat-shock proteins, and intracellular signaling factors (NFKBIA), i.e., factors also involved in COVID-19 pathology [128,129].

Further genetic polymorphisms related to malaria concern the gene encoding angiotensin-converting enzyme 2 (ACE2). ACE2 is characterized by a genetic deletion/insertion (D/I) polymorphism, which alters the concentration of the ACE I and D alleles, leading to reduced expression of ACE2. Analysis of African people’s descent reported several ACE1 and ACE2 polymorphisms. Regarding the presence of evolutionary adaptation related to malaria, different variants of the ACE2 receptor, which is used by SARS-CoV-2 to infect host cells, may protect populations and could potentially explain the heterogeneous incidence of COVID-19 among different African countries, e.g., Nigeria compared to South Africa [130,131,132].

Another approach attempts to identify potential targets for an immune response to SARS-CoV-2 by immune determinants’ shared identities with *Plasmodium falciparum*. Cross-reactivity was suggested through HLA-A*02:01 and subsequent CD8+ T-cell activation. The apparent immune dominant epitope conservation between SARS-CoV-2 (N and open reading frame (ORF) 1ab) and *Plasmodium falciparum* thrombospondin-related anonymous protein (TRAP) was hypothesized to provide shared antigens, providing immunity against virus infection to those previously infected with *Plasmodium falciparum* [127].

#### 3.5.5. Correlation of Malaria and the Expression of Duffy Receptor with COVID-19 Pathophysiology

A further interesting phenomenon in the context of malaria potentially having implications for COVID-19 incidence rates is the global expression pattern of the Duffy antigen receptor for chemokines (DARC), which is well known as the cell surface receptor used by the malaria parasite *Plasmodium vivax* (Figure 4b,c,d) to invade red blood cells (RBCs). DARC belongs to the minor blood group antigens with two immunologically codominant alleles, i.e., Fya and Fyb. Furthermore, the Fy-null phenotype (Fya–b–) results from a Fyb gene mutation in the DARC promoter region. These DARC polymorphisms form the basis for the four major Duffy blood group phenotypes: Fya, Fyb, Fya+b+, and Fya–b–. Variations in the Fy gene have been associated with phenotypic variation in susceptibility to *Plasmodium vivax* malaria. In fact, this gene has been shown to be one of the regions of the human genome that has been under the strongest selective pressure in our evolutionary history (selection coefficient: 4.3%). Accordingly, selection pressure has led to a very distinct expression pattern, with high Fya and Fyb antigen expression in areas without malaria, e.g., Caucasians (Fya 66% and Fyb 83%) and Asians (Fya 99% and Fyb 18.5%), but it is far less common in black people (Fya 10% and Fya 23%). The Fya–b– phenotype is present in two thirds of African American people but is very rare in Caucasians. In fact, the populations in West Africa do not express DARC on their erythrocytes and, as such, are resistant to *Plasmodium vivax* malaria [133].

In addition to being the cell surface receptor for *Plasmodium vivax* on red blood cells, DARC is a promiscuous receptor for various chemokines of both the CC and CXC classes, such as CXCL1 and IL-8 (CXCL8), regulated upon activation of normal T-expressed and secreted (RANTES/CCL5), monocyte chemotactic protein-1 (CCL2), neutrophil activating protein 2 and 3, epithelial neutrophil activating peptide-78 (CXCL5), and CXCL7/neutrophil-activating peptide 2, among others. DARC is homologous to chemokine G-protein chemokine receptors; however, unlike other seven-transmembrane chemokine receptors, Duffy Ag lacks the G-protein-coupling motif, which is essential for signal transduction. DARC is mainly expressed on the surfaces of erythroid cells and endothelial cells lining post-capillary venules in the kidneys, spleen, and neuronal cells. Until recently, DARC was considered a “silent” chemokine receptor, and its expression in erythrocytes was assumed to act as a sink or scavenger and regulate the bioavailability of several chemokines, including CXCL5, CXCL1, CXCL2, and CXCL8 [134]. Based on this assumed scavenger activity, a recent commentary discussed that the higher severity of COVID-19 in African Americans could correlate with the biological impact of an underlying Duffy null state [135].

However, the historically established hypothesis of erythrocyte Duffy Ag as a “sink” for circulating chemokines has been challenged by recent studies, which show that the in vivo role of Duffy Ag is more complex. In fact, various studies demonstrate that DARC mediates the effects of pro-inflammatory chemokines on endothelial cells lining post-capillary venules and neutrophil emigration to inflammation sites and mediating chemokine transcytosis. Furthermore, DARC was described to facilitate the movement of chemokines across the endothelium and to enhance IL-8/CXCL8-driven neutrophil recruitment into the lungs [136]. Another study determined whether Duffy Ag “loss-of-function” phenotypes are associated with alterations in plasma CXCL8 and CCL2 chemokine concentrations during the innate inflammatory response to LPS. Mice lacking erythrocyte DARC had higher MIP-2 and keratinocyte chemoattractant concentrations in the lung tissue vascular space but lower plasma chemokine concentrations associated with attenuated neutrophil recruitment into the airspaces, indicating that DARC alters soluble chemokine concentrations in blood and local tissue compartments and can enhance the systemic bioavailability of chemokines produced during local tissue inflammation. Since erythrocytes constantly transit through local tissue vascular beds, DARC may function as a chemokine reservoir, binding chemokines locally where concentrations are high during tissue inflammation and releasing chemokines systemically where the concentrations reach levels below the Kd value of chemokines for DARC. This, in effect, can enhance chemokine bioavailability in the systemic circulation produced during local tissue inflammation [137]. Furthermore, mice overexpressing DARC in the blood vessel endothelium were shown to have enhanced chemokine-induced leukocyte extravasation and contact-hypersensitivity reactions [138]. In a clinical trial, lipopolysaccharide-increased micro-particle-associated tissue factor (TF) pro-coagulant activation in Duffy antigen-negative Africans vs. Duffy-positive Caucasians was measured, showing a markedly reduced pro-coagulant response in healthy Duffy-negative subjects of African descent compared with Duffy-positive healthy Caucasians [139]. In a parallel study, Duffy-positive white subjects and Duffy-negative subjects of African descent were monitored after receiving an intravenous bolus of LPS. MCP-1 peak plasma levels were two-fold higher in Duffy-positive subjects than in Duffy-negative subjects. Erythrocyte-bound MCP-1, growth-related oncogene-alpha, and IL-8 increased 20- to 50-fold in Duffy-positive subjects. The authors concluded that Duffy antigen substantially alters chemokine concentrations in blood and does not have a protective effect during human endotoxemia [140]. Together, these data indicate that the Duffy receptor may have a significant—so far not completely understood—impact on COVID-19-associated chemokine biodistribution and bioavailability, with an impact on inflammatory cell migration and extravasation [141]. The high frequency of DARC negativity among people from the sub-Saharan region of Africa can be assumed to provide an advantage in limiting chemokine transport to sites distant from the initial SARS-CoV-2 infection.

#### 3.5.6. Coverage of Malaria Treatment with Artemisinin and Its Effect on the COVID-19 Triggered Cytokine Storm

Regarding the second possibility, therapies used for malaria treatment or prophylaxis could be responsible for the observed inverse correlation between incidences of malaria and COVID-19 reported cases. In this context, chloroquine and hydroxychloroquine prompted a wide initial interest in repurposing for the treatment of COVID-19. Meanwhile, the results of large WHO trials and a Recovery study have shown rather disappointing results, and a recent study has suggested that chloroquine and hydroxychloroquine might even be harmful in hospitalized COVID-19 patients [142]. Meanwhile, the WHO has withdrawn its recommendation for the use of chloroquine and hydroxychloroquine.

According to the WHO website, the best available treatment, particularly for *Plasmodium falciparum* malaria, is artemisinin-based combination therapy (ACT) (Figure 4e,f). The mode of action makes artemisinin—the active component of *Artemisia annua* L. (qinghao, sweet wormwood)—a very interesting candidate for drug repurposing for COVID-19. Dihydroartemisinin (DHA), a clinically relevant compound, was shown to kill parasites via a two-pronged mechanism, causing protein damage and compromising parasite proteasome function. As a consequence, unprocessed proteasome substrates, i.e., unfolded/damaged and polyubiquitinated proteins, accumulate and activate the ER stress response. Other specific proteasome inhibitors caused a similar build-up of polyubiquitinated proteins, leading to parasite killing. Notably, other anti-malaria drugs, such as chloroquine, did not show proteasome inhibition [143].

Inhibition of proteasome activity is known to critically affect the nuclear translocation of the transcription factor NF-κB [42], and this was shown also for artemisinin, which inhibited NF-κB -regulated nitric oxide synthase and LPS or cytokine-induced activation of NF-κB in human astrocytoma cells [144] or in microglial cells [145]. 

Furthermore, Wang et al. demonstrated that artemisinin inhibits the secretion and mRNA levels of TNF-α, IL-1β, and IL-6 in a dose-dependent manner in phorbol 12-myristate 13-acetate (PMA)-induced THP-1 human monocytes. They also found that the NF-κB-specific inhibitor Bay 11-7082 inhibited the expression of these pro-inflammatory cytokines, suggesting that the NF-κB pathway is involved in decreased cytokine release. Artemisinin impeded the phosphorylation of IKKα/β, the phosphorylation and degradation of IκBα, and the nuclear translocation of the NF-κB p65 subunit [146]. Similarly, pretreatment of cells with artemisinin prevented the TNF-α-induced expression of NF-κB target genes, such as invasion (MMP-9), angiogenesis (VEGF), and major inflammatory cytokines (TNF-α, iNOS, and MCP1) [147]. Importantly, as summarized in a recent review, NF-κB activation was proposed to have a central role in the COVID-19-induced cytokine/chemokine storm [42], and the treatment of COVID-19 patients with artesimin based on its NF-κB and cytokine production inhibiting activity was proposed in a recent paper by Tang et al. [148].

## 4. Integrated Mechanistic Model for Lower COVID-19-Associated Mortality in the Sub-Saharan African Region

Whereas Europe and North and South America have been heavily affected by high infection and death rates, unexpectedly significantly lower mortality has been reported in Africa, particularly in the sub-Saharan region. This is in striking contrast to what was to be expected considering Africa’s generally weak health system, poor access to medical supplies, population crowding in African cities, and poverty and unhygienic conditions. A mechanistic explanation for this “African paradox” may be very helpful for the understanding of COVID-19 disease and therapeutic approaches.

**Figure 5:**
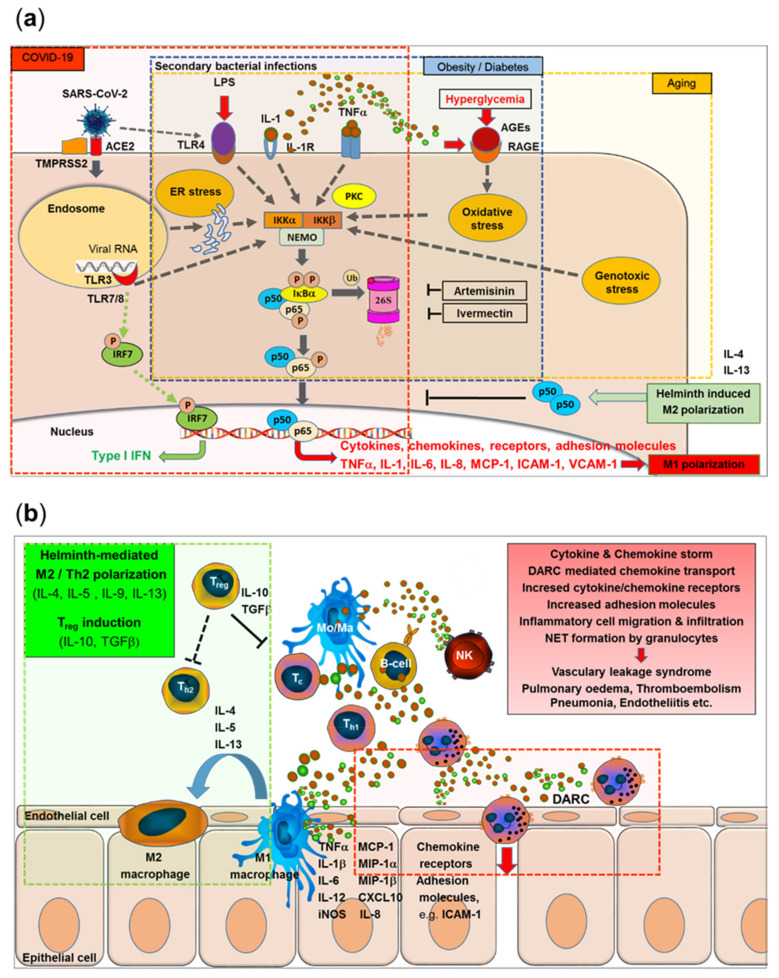
(**a**). SARS-CoV-2 binds to the ACE-2 receptor, followed by spike protein cleavage, membrane fusion, and endocytosis. RNA of single-stranded RNA viruses activates the Toll-like receptors TLR7 and TLR8. Double-stranded RNA intermediates are recognized by TLR3. TLR activation triggers transcription of the interferon-regulator factor (IRF) family, but also the activation of IKK (IκB kinases), phosphorylation, ubiquitination, and proteasomal degradation of the cytoplasmic inhibitor factor IκBα, and release of the NF-κB (p50/p65) to enter the nucleus. The final sequence of NF-κB activation is shared with multiple cytokine receptor- and TLR-mediated signal cascades, e.g., binding of TNFα or IL-1 to their receptors or binding of LPS to TLR4. Additionally, SARS-CoV-2 leads to TLR4-mediated and ER stress-induced NF-κB activation. NF-κB activation triggers gene expression for multiple pro-inflammatory cytokines and chemokines (with a typical M1/Th1 polarization pattern), adhesion molecules, and acute phase proteins. Released cytokines feed into accelerating feedback loops. In contrast, interferon-response factor (IRF)-related responses are largely independent of NF-κB translocation (**a**, red transparent area). Metabolic disorders, e.g., obesity and diabetes, lead to chronic NF-κB pre-activation via hyperglycemia-induced IKKβ overexpression, Protein kinase C (PKC) activation, and advanced glycosylation end products (AGEs) that bind to receptors for advanced glycosylation end products (RAGE) on vascular smooth muscle cells, leading to oxidant stress. NF-κB triggered cytokine (TNFα, IL-1β) expression feeds into accelerating feedback loops via TNFα/IL-1β receptor-triggered NF-κB activation and increased expression of RAGE (**a**, blue transparent area). Aging-related genotoxicity, inflammation, ER stress, and oxidative stress stimulate the NF-κB pathway. An “inflamm-aging”-associated dysregulation of cytokine homeostasis feeds into auto amplifying loops, enhancing the COVID-19-triggered acute NF-κB activation storm (**a**, yellow transparent area). The anti-helminth drug ivermectin and the anti-malaria drug artemisinin inhibit the NF-κB pathway at various steps, with artemisinin (a proteasome inhibitor) blocking the degradation of IκBα and the nuclear translocation of NF-κB. (**b**). Helminth infection induces M2/Th2 predisposition, Treg, and immunosuppressive cytokines IL-10 and TGFβ (**b**, green transparent area), which can counteract SARS-CoV-2-triggered NF-κB-mediated M1/Th1 polarization (**a**,**b**, green inserts). Duffy receptor-mediated transport and transcytosis of chemokines can enhance the migration, adhesion, and extravasation of macrophages and neutrophils even at sites distant to the initially SARS-CoV-2-infected cells (**b**, red transparent area), leading to the activation and damage of endothelial cells, vascular leakage, pulmonary edema, thromboembolism, and multiorgan endotheliitis (**b**, red insert). This effect may be reduced in geographical areas with prevalent DARC negativity. (**a**) represents a modification of Figure 1 previously published in https://pubmed.ncbi.nlm.nih.gov/33362782/ accessed on 8 September 2021.

Here, we present a model that integrates the major immunological and epidemiological specifics reported for Africa with the emerging picture of immune pathological mechanisms characteristic of COVID-19 and provides a mechanistic explanation for the African paradox of low COVID-19 morbidity and mortality. The various lines of this model merge at the NF-κB signaling pathway as the central point, which may help to identify new targets for drug development or repurposing existing drugs with known mechanisms of action (Figure 5a,b).

SARS-CoV-2 binds to its receptor, i.e., the angiotensin-converting enzyme-related carboxypeptidase-2 (ACE-2) receptor, by the RBD of the spike (S) protein, followed by cleavage, membrane fusion, and endocytosis into the host cell. Activation of Toll-like receptors, including TLR7, TLR8, and TLR3, leads to activation of the transcription of the interferon-regulator factor (IRF) family and antiviral responses but also to the activation of IKK (IκB kinases) followed by phosphorylation and ubiquitination of cytoplasmic inhibitor factor IκBα and its degradation by the 26S proteasome. Thereby, NF-κB is released from IκBα and can then enter the nucleus and initiate the transcription of various genes coding for pro-inflammatory proteins, such as cytokines, chemokines, adhesion molecules, and growth factors. Importantly, this final sequence of NF-κB activation is shared with a range of cytokine receptor- and Toll-like receptor-mediated signal cascades, including binding of TNFα or IL-1 to their receptors or binding of LPS (e.g., from secondary bacterial infections) to TLR4. Furthermore, SARS-CoV-2-induced TLR4-mediated NF-κB activation and ER stress-induced NF-κB activation have been reported. Excessive NF-κB activation triggers gene expression for a broad range of pro-inflammatory cytokines and chemokines (with a typical M1/Th1 polarization pattern), adhesion molecules, and acute phase proteins. Released cytokines feed into accelerating positive feedback loops, leading to inflammatory cell activation and infiltration, vascular leakage syndrome, pulmonary edema, and pneumonia (Figure 5a, red transparent area). In contrast, interferon-response factor (IRF)-related responses were largely independent of NF-κB translocation.

Furthermore, typical metabolic disorders, such as obesity and diabetes, lead to chronically elevated levels of NF-κB pre-activation via hyperglycemia-induced IKKβ overexpression, increased Protein kinase C (PKC) activity, or the formation of advanced glycosylation end products (AGEs) binding to specific receptors, i.e., receptors for advanced glycosylation end products (RAGE) on vascular smooth muscle cells, leading to oxidant stress, i.e., reactive oxygen species (ROS). NF-κB activation-triggered cytokine (TNFα, IL-1β) expression feeds into auto amplifying loops via TNFα/IL-1β receptor-triggered NF-κB activation and increased expression of RAGE (Figure 5a, blue transparent area).

Furthermore, aging-related genotoxicity, inflammation, ER stress, and oxidative stress have been shown to stimulate the NF-κB pathway. An “inflamm-aging”-associated dysregulation of cytokine homeostasis will feed into accelerating positive feedback loops, enhancing the COVID-19-triggered acute NF-κB activation storm (Figure 5a, yellow transparent area).

The anti-helminth drug ivermectin and the anti-malaria drug artemisinin, both with wide coverage in the sub-Saharan helminth and malaria belt in Africa (see Figure 4f), inhibit the NF-κB pathway at various steps, with artemisinin also being a proteasome inhibitor, inhibiting the degradation of cytoplasmic inhibitor factor IκBα and the nuclear translocation of NF-κB.

Furthermore, helminth infection-induced M2 polarization may counteract NF-κB-induced M1 polarization by IL-4-mediated effects and induction of the negative NF-κB regulator p50 (Figure 5a,b green inserts). Helminth infection not only leads to typical M2/Th2 predisposition but also induces immune regulatory mechanisms and release of the immunosuppressive cytokines IL-10 and TGFβ (Figure 5b, green transparent area). 

SARS-CoV-2-triggered NF-κB-mediated M1/Th1 polarization leads to increased release of pro-inflammatory cytokines (such as TNFα, IL-1β, IL-6, and IL-12), inducible reactive nitrogen species (iNOS), chemokines (MCP-1, MIP1α, MIP1β, CXCL10, and IL-8), and their receptors, together with increased expression of adhesion molecules such as ICAM-1 and VCAM-1. Duffy receptor-mediated transport and transcytosis of chemokines may further enhance the attraction, adhesion, and extravasation of innate immune cells, such as macrophages and neutrophils, further enhancing pro-inflammatory effects even at sites distant to the initial SARS-CoV-2-infected cells (Figure 5b, red transparent area), leading to the activation and damage of endothelial cells, vascular leakage, pulmonary edema, thromboembolism, and multiorgan endotheliitis (Figure 5b, red insert). This process may be less pronounced in geographical regions with high DARC negativity prevalence in the population.

## 5. Discussion

To develop a comprehensive mechanistic explanation of the African paradox and to correlate with the underlying molecular mechanisms in COVID-19, we addressed various socioeconomic, epidemiological, genetic, and immunological factors and combined them into one integrated model on the basis of known and anticipated immunological and pathophysiological mechanisms underlying the COVID-19 disease. Among the factors discussed, i.e., young average age, low number of nursing homes for elderly individuals, lower comorbidities, previous experience in epidemic control from tackling other infectious diseases, warm climate, genetic disposition (HLA variability, different cytokine responsiveness), epidemiology, and pre-exposure to different pathogens, we have identified several factors with a particularly high potential impact on COVID-19-related morbidity and mortality and their correlation with immune pathological mechanisms that seem to be central in the COVID-19 disease. From the present review, the following factors appear to be particularly relevant to the “African paradox”: (1) the young age of the African population, (2) the low incidence of obesity and diabetes, (3) factors related to high helminth infection incidence, (4) factors related to high malaria incidence with a potential major effect of Duffy receptor negativity. Notably, all factors are linked to the NF-κB signal transduction pathway.

The highest impact may be expected from population characteristics, i.e., the young average age of the African population and the low incidence of comorbidities typically associated with severe clinical COVID-19 pathology, i.e., obesity and diabetes and other diseases with metabolic syndrome. Notably, all have been found to be associated with chronically higher levels of pro-inflammatory cytokines and chemokines and a general chronic immune activation status. Although the initial pathways may differ for the selected comorbidities, all have been demonstrated to activate the NF-κB pathway as one central pro-inflammatory pathway.

Similarly, aging is associated with elevated levels of local and systemic pro-inflammatory cytokines, creating a chronic condition of inflammation in aged subjects, named “inflamm-aging”, with elevated levels of cytokines and chemokines. Therefore, it is likely that, similar to obesity and diabetes, dysregulation of cytokine homeostasis in the “inflamm-aging” phenomenon may play a critical role in the risk of a cytokine storm and subsequently acute respiratory distress syndrome (ARDS) or multiorgan failure in particularly elderly persons after SARS-CoV-2 infection. Aging-related genotoxicity, inflammation, ER stress, and oxidative stress have been shown to stimulate the NF-κB pathway. This chronic preactivation state enhances the acute NF-κB activation found for COVID-19, resulting in exuberant pro-inflammatory responses followed by the natural phenomenon of immune exhaustion [149,150,151,152,153]. Accordingly, lowering this pre-activation of NF-κB by using natural alimentary components (curcuma, garlic, onion, green tee, oregano, thyme) with known NF-κB inhibitory activity could provide some ameliorating effect. For the treatment of acute stages of COVID-19, clinical studies testing clinically proven NF-κB inhibitors (e.g., aspirin) for their application in COVID-19 therapy might be a highly promising approach, overcoming the limitations of approaches targeting single cytokines [42]. For clinical studies, the right dose regimens, e.g., “anti-inflammatory” high doses of aspirin vs. “anticoagulation” low doses, and optimized start/duration of treatment will have to be evaluated [154].

A second major topic with regard to the lower morbidity and mortality of COVID-19 in Africa is epidemiological factors. High incidences of helminth infections and malaria in subtropical–tropical regions could have an impact on COVID-19-associated morbidity and mortality in Africa. Helminth-pre-induced type 2 responses may counterbalance the typical massive M1/Th1 polarization of COVID-19. Furthermore, a commonly applied anti-helminth drug, i.e., ivermectin, was found to inhibit the release of pro-inflammatory cytokines and to inhibit NF-κB activation. However, its potency for the treatment of COVID-19 has recently been discussed controversially.

A further important factor may be concluded from the coincidence of the geographical distribution of lower COVID-19-associated morbidity and mortality in the sub-Saharan area with an overlapping geographical region of malaria endemic. The epidemiological phenomenon due to the high genetic selection pressure by this devastating infectious disease and the coverage by specific anti-malaria drugs could have an ameliorating effect on COVID-19 in these regions. From the present perspective, we hypothesize that the global expression pattern of the Duffy antigen receptor for chemokines (DARC), with a high frequency in the Duffy-negative population, particularly in the subtropical regions of Africa, as a result of the high selection pressure brought on by *Plasmodium vivax* could be one of the factors contributing to the lower COVID-19 incidence in Africa. Notably, the distribution of Duffy receptor expression does not follow the distribution of *Plasmodium falciparum* because—in contrast to *Plasmodium vivax—Plasmodium falciparum* uses multiple receptors for cellular entry. Recent studies demonstrate that DARC can mediate the effects of pro-inflammatory chemokines on endothelial cells lining post-capillary venules as well as neutrophil emigration to inflammation sites and mediate chemokine transcytosis [134] to facilitate the movement of chemokines across the endothelium and to enhance neutrophil recruitment into the lungs [136], thus enhancing chemokine-induced leukocyte extravasation.

In this context, it should be noted that in contrast to the narrow interpretation of the term COVID-19-induced “cytokine storm”, this immune pathological complex includes cytokines and multiple chemokines, which have been shown to correlate with the severity of COVID-19 symptoms [34]. Chemokines are instrumental in attracting innate and immune cells, particularly monocytes and neutrophil granulocytes, which are expected to impose a major impact on COVID-19-associated pathology [155]. Chemokine-mediated attraction is followed by the adhesion of inflammatory cells to the endothelial cells of the blood vessels in various organs and migration and extravasation into the sub-endothelial space [47,141]. Damage of the endothelial layers of blood vessels, vascular leakage, and edema can be the consequences of increased attraction, adhesion, and migration even at sites distant to the primary infection of SARS-CoV-2. In this context, the Duffy receptor may have a significant—so far not completely understood—impact on COVID-19-associated immunopathology.

Finally, coverage in malaria-endemic areas, particularly in Africa, with the anti-malaria drug artemisinin may have an additional ameliorating effect on COVID-19 severity. Artemisinin was demonstrated to inhibit NF-κB pathway activation by inhibiting proteasome activity, resulting in a significant reduction in the release of pro-inflammatory cytokines and chemokines.

Conclusively, a combined effect of young age population with lower comorbidities, epidemiological effects from highly incident helminth parasite infections and malaria endemic infection, together with a high coverage of anti-helminth and anti-malaria drugs with pronounced anti-inflammatory, i.e., NF-κB-inhibiting activity, may be responsible for the surprisingly low COVID-19-associated mortality rate in the sub-Saharan region of Africa. Insight into epidemiological and pathophysiological/immunological correlations may help in the selection of promising therapeutic approaches and targets for the treatment of COVID-19, including repurposing existing drugs. In this respect, testing known NF-κB inhibitors [156,157] and their repurposing for the treatment of COVID-19 may be a promising approach [42].
